# The impact of the malaria centre program on malaria incidence in Papua Province

**DOI:** 10.1016/j.puhip.2025.100625

**Published:** 2025-05-15

**Authors:** Eni Setianingsih, Eny Sulistyaningrum

**Affiliations:** a Development Economics Program, Universitas Gadjah Mada, Indonesia; b Department of Economics, Universitas Gadjah Mada, Indonesia

**Keywords:** Impact evaluation, Malaria centre, Fixed effects, Annual parasite incidence

## Abstract

**Objective:**

The goal of eliminating malaria in Indonesia by 2030 faces significant challenges, particularly due to the stagnation of malaria cases in recent years. This issue is exacerbated by the high concentration of cases in Eastern Indonesia, with Papua Province alone contributing to 86 % of the national malaria cases. To address this, the Malaria Centre Programme has been implemented to eradicate malaria in highly endemic regions. This study aims to evaluate the impact of the malaria centre program on the prevalence of malaria in Papua.

**Study design:**

a mixed-method approach.

**Method:**

First, fixed effects analysis effectively evaluates program impacts shaped by pre-existing attributes. Second, in-depth interviews help identify potential impacts, analyze mechanisms, and assess benefits to beneficiaries.

**Result:**

The findings indicate that the malaria centre program has successfully reduced the annual parasite incidence (API) in Papua Province by 31.81 cases per 1000 population. Additionally, controlling for healthcare facilities, especially the ratio of community health centres, significantly lowers malaria incidence by 430.695 cases. However, the availability of hospitals does not significantly impact malaria incidence in the province.

**Conclusion:**

The malaria centre program has had a significant impact on reducing malaria incidence in Papua Province. Implementing the program has effectively lowered malaria cases in several regencies, including Keerom, Nabire, Boven Digoel, and Jayapura.

## 1 Introduction

Malaria, caused by the Plasmodium parasite, is a leading global killer and significantly reduces productivity in affected individuals [1]. WHO data from 2015 shows 438,000 deaths from 214 million cases, with 88 % of these fatalities in Africa, followed by Southeast Asia (10 %) and the Eastern Mediterranean (2 %) [2]. Predominantly found in tropical and subtropical areas, such as Indonesia, the WHO ranks Indonesia second in Southeast Asia for malaria cases after India [3]. Although Indonesia's annual parasite incidence declined from 2010 to 2014, progress stagnated between 2015 and 2019. Malaria is mainly concentrated in Eastern Indonesia, with 27 regencies and cities showing high endemicity. In 2019, Papua Province accounted for 86 % of the country's malaria cases, the highest in Indonesia [4]. Papua consistently has the highest malaria incidence in the country [5].

Malaria control strategies have effectively reduced incidence in many countries. In Zambia, house screening interventions lowered malaria prevalence and sick days, improving workforce productivity [6]. However, long-lasting insecticidal nets (LLIN) for children in Ghana showed no significant impact [7]. Pinder et al. (2016) found that combining long-lasting insecticidal nets (LLINs) with indoor residual spraying (IRS) is crucial for reducing malaria cases [6]. In Zanzibar, reactive case detection (RCD) reduced transmission but was insufficient for elimination [8]. Artemisinin-based combination therapy (ACT) also lowered malaria incidence [9].

The PAMAFRO program in Peru, involving LLIN distribution, enhanced diagnostics, and environmental management, achieved a 78 % reduction in malaria incidence, though results varied by region and species [10]. Research by Gelatas et al. (2020) in Mozambique found that combining surveillance, LLINs, IRS, and mass drug administration (MDA) significantly reduced malaria prevalence [11]. In North Maluku, Indonesia, the malaria centre program cut cases by 48 % from 2003 to 2008, though there was a 15.29 % increase in 2010 [12].

The Indonesian government is targeting malaria elimination by 2030 through Minister of Health Decree No. 293/MENKES/SK/IV/2009, aligning with the global Sustainable Development Goals (SDGs). The strategy involves establishing a malaria centre to coordinate programs across provincial and district levels, serving as the hub for prevention and resource development [13]. The flagship program, first implemented in South Halmahera, North Maluku, an area with high mortality in 2003, has significantly reduced malaria deaths [14].

Malaria centre programs implement comprehensive strategies to combat malaria, including vector control measures such as the distribution of insecticidal nets, indoor residual spraying (IRS), and community empowerment in environmental management to reduce breeding sites. Additionally, these programs emphasize early case detection through rapid diagnostic tests (RDT) and mass blood surveys (MBS), followed by prompt and effective patient treatment. To enhance long-term malaria management, they focus on strengthening healthcare capacity through training and resource development. Furthermore, these initiatives promote cross-sectoral collaboration by engaging government agencies, non-governmental organizations (NGOs), and local communities to ensure a coordinated and sustainable approach to malaria prevention and control [12].

**Fig. 1:**
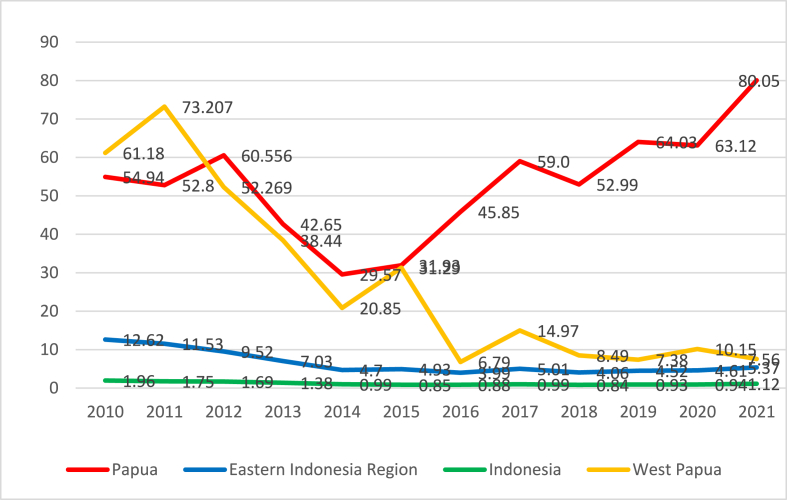
Annual parasite incidence in Indonesia, eastern Indonesia region, west Papua Province, and Papua Province (Ministry of health Indonesia).

Despite such intervention efforts, malaria remains a persistent challenge in Papua Province, Indonesia. Fig. 1 shows that the annual parasite incidence (API) in Papua Province surpasses the malaria prevalence in Indonesia, the Eastern Indonesia Region, and West Papua Province. Although there was a decline in malaria incidence in Papua from 2011 to 2014, there was a subsequent increase from 2015 to 2021. This rising incidence presents a major challenge to malaria elimination efforts in Papua. The high number of regencies and cities with high endemicity significantly contributes to the increased malaria prevalence in Papua Province.

The high endemicity of malaria is a key factor in implementing the malaria centre program, which began in 2013 in areas such as Mimika, Biak Numfor, and Jayapura City. It was followed by Keerom in 2018 and Jayapura Regency, Boven Digoel, and Nabire in 2019. Despite this, some regions with high endemicity have not yet adopted the program. In Papua Province, the program has been established in 7 regencies/cities. Yet, the province still has the highest malaria incidence in Indonesia, with a rate of 64.03 cases per 1000 population as of 2019.

This study evaluates the impact of the malaria centre program on malaria incidence in Papua, where the annual parasite incidence remains high. Addressing a gap in the literature, which has mainly focused on Africa. It offers a comprehensive evaluation of the program in Papua, unlike previous studies that did not assess its effectiveness in North Maluku. Using a mixed-methods approach, this study seeks to provide empirical evidence on the program's impact, offering insights to improve malaria control policies.

## 2 Methods

### 2.1 Data

This study uses panel data on annual parasite incidence (API) as the dependent variable and the presence of a malaria centre as the independent variable. Covariates include healthcare facilities (hospital and health centre ratios) and socioeconomic factors like school enrollment rates, GRDP per capita, population size, and region. Geographical factors such as area size and altitude are also considered for regencies/cities in Papua Province.

Data on APIs, hospitals, and health centres were sourced from the Papua Provincial Health Office, while data on the malaria centre program were obtained from the Ministry of Health Indonesia and local government websites. Additional data on population size, GRDP per capita, enrollment rates, area size, and altitude were obtained from the Papua Provincial Statistics and regional categories from the Ministry of Villages.

The quantitative data collection process utilized secondary data from the years 2018–2021, comprising a total of 116 observations. The number of observations was determined based on data availability, which was sourced from the Papua Provincial Health Office.

The qualitative data collection process was conducted through in-depth interviews in Mimika Regency. The selection of this location was based on the complexity of malaria cases in the area and its role as one of the first regencies to implement the Malaria Centre program. A purposive sampling technique was employed to select interview respondents, involving three groups: malaria centre programme implementers, the community, and the government. This approach aimed to obtain a comprehensive perspective on the program's implementation while also enabling a thorough cross-verification of its execution and impact.

### 2.2 Study Design

This study evaluates the impact of the malaria centre on malaria incidence in Papua Province using a fixed effects quantitative method and a qualitative approach to analyze the program's mechanisms, potential impacts, and benefits to beneficiaries [15]. A quantitative approach alone can't fully evaluate outcomes or counterfactuals and may introduce statistical biases. Thus, a mixed-methods approach, blending qualitative and quantitative methods, offers a more comprehensive assessment of a program's effectiveness [15]. A qualitative approach was conducted through in-depth interviews after obtaining the estimation results from the quantitative study. This approach aims to achieve a more comprehensive understanding and provide a detailed explanation of the phenomena related to the implementation of the Malaria Centre program in Papua Province.

In policy impact evaluations, there is often a link between policy variables and unobserved characteristics [16]. The fixed effects model addresses this by eliminating unobserved heterogeneity [17]. The fixed effects model is ideal for social epidemiology, which is often complicated by contextual, behavioral, and attitudinal factors [18]. It treats the variable of interest as a traditional experiment's "treatment" effect [17]. This approach is especially effective for analyzing program participation impacts influenced by pre-existing attributes that also affect outcomes [16]. The study's model equation [6,7,19]:

(1) ***API*_*it*_** = β**_*0*_ +*β*_*1*_*MC*_*it*_** +***β*_*2*_*RS*_*it*_** +***β*_*3*_*Puskes*_*it*_** + ***β*_*4*_*APSSD*_*it*_**+***β*_*5*_*APSSMP*_*it*_**+***β*_*6*_*APSSMA*_*it*_** +***β*_*7*_*lPop*_*it*_** +***β*_*8*_*lPDRB*_*it*_** +***β*_*9*_*Reg*_*it*_** + ***β*_*10*_*lArea*_*it*_** + ***β*_*11*_*lAlt*_*it*_** + ***γ*_*t*_** + ***u*_*it*_**

The subscript i denotes the observed regency/city unit, and t represents the observation period. *API*_*it*_ is the malaria incidence rate per 1000 population. *MC*_it_ is a binary variable that equals 1 for regencies/cities implementing a malaria centre program and 0 otherwise. *RS*_*it*_ is the ratio of hospitals per 1000 population, and *Puskes*_*it*_ is the ratio of health centres per 1000 population. *APSSD*_*it*_, *APSSMP*_*it,*_ and *APSSMA*_*it*_ represent the decimal form of the percentage of enrolment rates at the elementary school, junior high, and senior high school levels. *lPop*_*it*_ is the logarithmic form of the population size. *lPDRB*_*it*_ is the logarithmic form of the Gross Regional Domestic Product. *Reg*_*it*_ is a binary variable for the regional category, with a value of 2 for regencies/cities in the developing category, 1 for regencies/cities in the underdeveloped category, and 0 for regencies/cities in the severely underdeveloped category. *lArea* is the logarithmic form of the area in square kilometers (km^2^). *lAlt*_*it*_ is the logarithmic form of the altitude in meters above sea level (masl). ***γ***_t_ represents year-fixed effects (year FE), and *u*_*it*_ is the error term.

## 3 Results

### 3.1 Statistics analysis

The descriptive analysis presented in Table 1 the mean annual parasite incidence (API) in Papua Province from 2018 to 2021 was 68.440, placing the province in the high endemicity category II. The mean annual parasite incidence in Papua Province significantly surpasses the national average of 0.83 recorded in 2020 (Statistics Indonesia, 2021). The malaria centre program in Papua Province currently has a coverage proportion of 21.5 %. This proportion is considered low given the high incidence of malaria spread across most regencies/cities in Papua. However, the availability of healthcare facilities such as hospitals and health centres remains relatively low. Table 1 shows that the mean hospital ratio per 1000 population is 0.014, and the mean health centre ratio is 0.160. The health centres ratio in Papua Province is the lowest compared to the Indonesia health centres ratio [20].

**Table 1:** Summary statistics.

Variables	Mean	Standard Deviation	Min	Max
Annual Parasite Incidence	68.440	110.962	0.021	383.012
Malaria centre	0.215	0.412	0	1
Hospital ratio per 1000 Population	0.0148	0.011	0	0.049
Health centre ratio per 1000 Population	0.160	0.099	0.035	0.514
Primary school enrolment rate (%)	83.724	13.323	51.610	98.030
Junior high school enrolment rate (%)	79.981	16.895	29.030	98.070
Senior high school enrolment rate (%)	61.388	18.456	22.550	97.120
GRDP per capita in thousands of Indonesian Rupiah	24,469.958	18,136.989	4265.889	77,482.389
Population size	132,422	85,965	20,018	404,004
Area size (km^2)^	10,887.120	10,547.010	537,390	47,406.900
Altitude (mdpl)	722,940	852,756	4786	2303.590
Region				
Underdeveloped regions	0.362	0.482	0	1
Developing regions	0.034	0.183	0	1

An analysis of socio-economic educational characteristics shows that in Papua Province, school enrollment rates are highest at the elementary level (83.73 %) but decline at junior high (79.98 %) and further at senior high (61.38 %). Papua's enrollment rates are consistently below the national average, highlighting persistent challenges in educational participation.

Additionally, the Gross Regional Domestic Product (GRDP) per capita from 2018 to 2021 in Papua Province averaged Rp24,469,958. The average PDRB per capita in Papua is lower than the Indonesian mean GRDP per capita in 2021 (Statistics Indonesia, 2021). The lower Gross Regional Domestic Product (GRDP) per capita in Papua Province reflects its underdeveloped economic conditions.

Papua Province's demographic diversity is evident in its regencies/cities, with populations ranging from 20,018 to 404,004 and an average of 132,422. High population densities strain healthcare systems and limit malaria control resources. The average land area is 10,887.12 km^2^, with the largest at 47,406.9 km^2^, challenging access, especially in remote areas. At an average elevation of 722.94 m, high malaria incidence in both highland and lowland regions complicates control efforts.

In terms of development, 60.32 % of regencies are severely underdeveloped, creating significant challenges for growth and malaria control. Only 3.45 % are classified as developed, while 36.20 % are underdeveloped. More significant underdevelopment correlates with increased difficulty in combating malaria.

### 3.2 Description of Malaria Endemicity distribution in Papua Province

Papua Province, characterized by its high levels of malaria prevalence, illustrates the intricate dynamics of malaria cases. The incidence of malaria has demonstrated fluctuations from 2018 to 2021. The persistent high incidence of malaria, resistant to decline, underscores the complexity of the challenges faced in addressing this disease. The elevated prevalence of malaria in Papua Province is closely linked to the widespread distribution of regencies/cities in Papua characterized by high endemicity status.

**Fig. 2:**
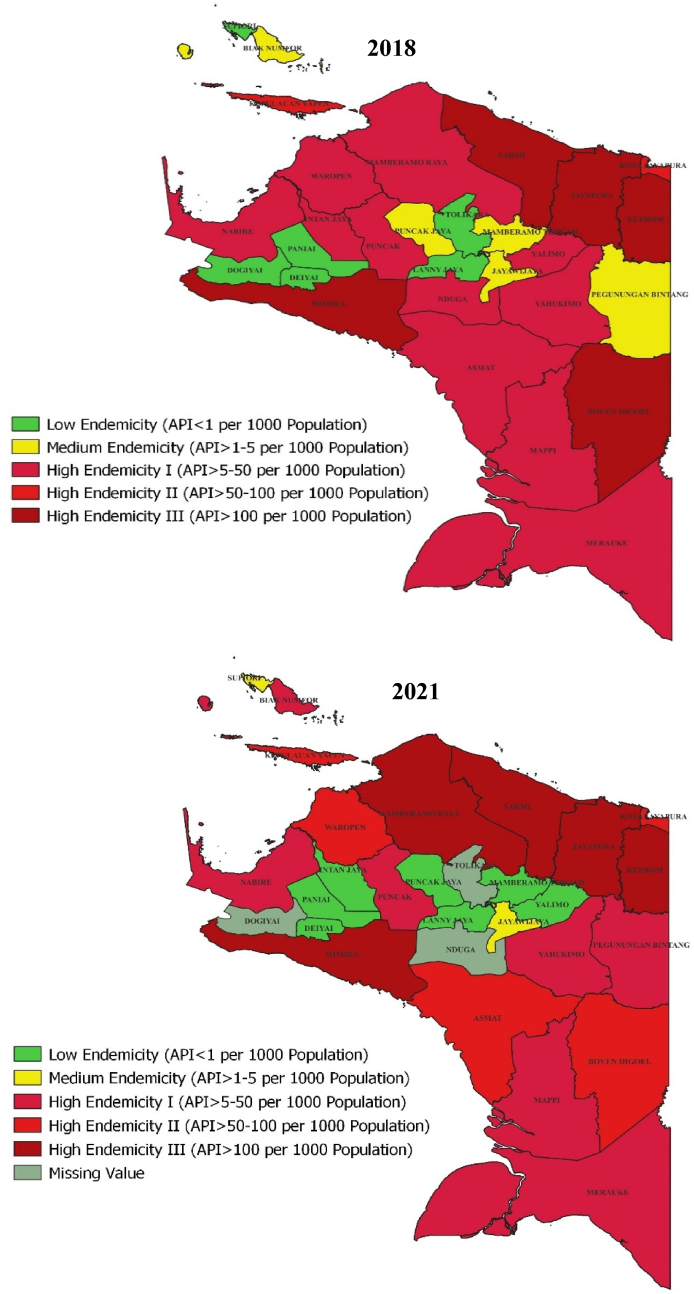
Map of malaria endemicity in Papua province in 2018 and 2021 (Papua provincial health office).

Fig. 2 shows that in 2018, Mimika, Boven Digoel, Keerom, Jayapura, and Sarmi were level III high endemicity areas, with annual parasite incidence (API) exceeding 100 cases per 1000 population. Keerom had the highest incidence at 381.73 cases per 1,000, emphasizing the need for targeted interventions. In Papua Province, malaria is mainly level I high endemicity (API 5–50 cases per 1000) across 11 regencies/cities. 5 regencies are classified as having moderate endemicity, while 6 regencies exhibit low endemicity, predominantly located in high-altitude regions above 700 m above sea level (masl). High-endemicity areas are generally in low to moderate altitudes (below 700 masl) with temperatures of 25 °C–29 °C, while lower-endemicity regions are in cooler, high-altitude areas (18 °C–20 °C).

Severe malaria outbreaks can occur in regions with average temperatures ranging from 18 °C to 38 °C, while optimal malaria transmission occurs at 28 °C [3]. Normal or warm temperatures provide optimal conditions for the development of Anopheles mosquito vectors responsible for malaria transmission. It is important to note that the lower the elevation of an area, the higher the risk of malaria cases [21].

In 2021, Papua Province showed significant progress in malaria control compared to 2018. High endemicity level I regencies decreased from 11 to 6, while 5 regencies remained at level III. Boven Digoel saw a reduction in cases, while Mamberamo Raya's cases increased to level III. The number of low endemicity regencies increased to 7, reflecting a decline in malaria incidence in several areas. These changes underscore the factors driving reduced malaria cases and support more targeted control strategies.

### 3.3 Description of the malaria centre program and annual parasite incidence in Papua Province

The efforts in malaria control involve implementing malaria centre programs in regions with high endemicity status. The establishment of malaria centres takes into consideration various factors such as endemic areas, regions facing complex malaria issues, allocation of local government revenue and expenditure for malaria control activities that are still inadequate or unavailable, and low community involvement in malaria control efforts [13].

The implementation of this program varies by region, depending on local readiness, capacity, and malaria complexity. Adapting programs to local contexts and fostering collaboration among local governments, health institutions, and stakeholders is crucial for optimal malaria prevention and control. Regencies/cities with high malaria incidence, good accessibility, and adequate facilities are typically the ones adopting this program.

**Fig. 3:**
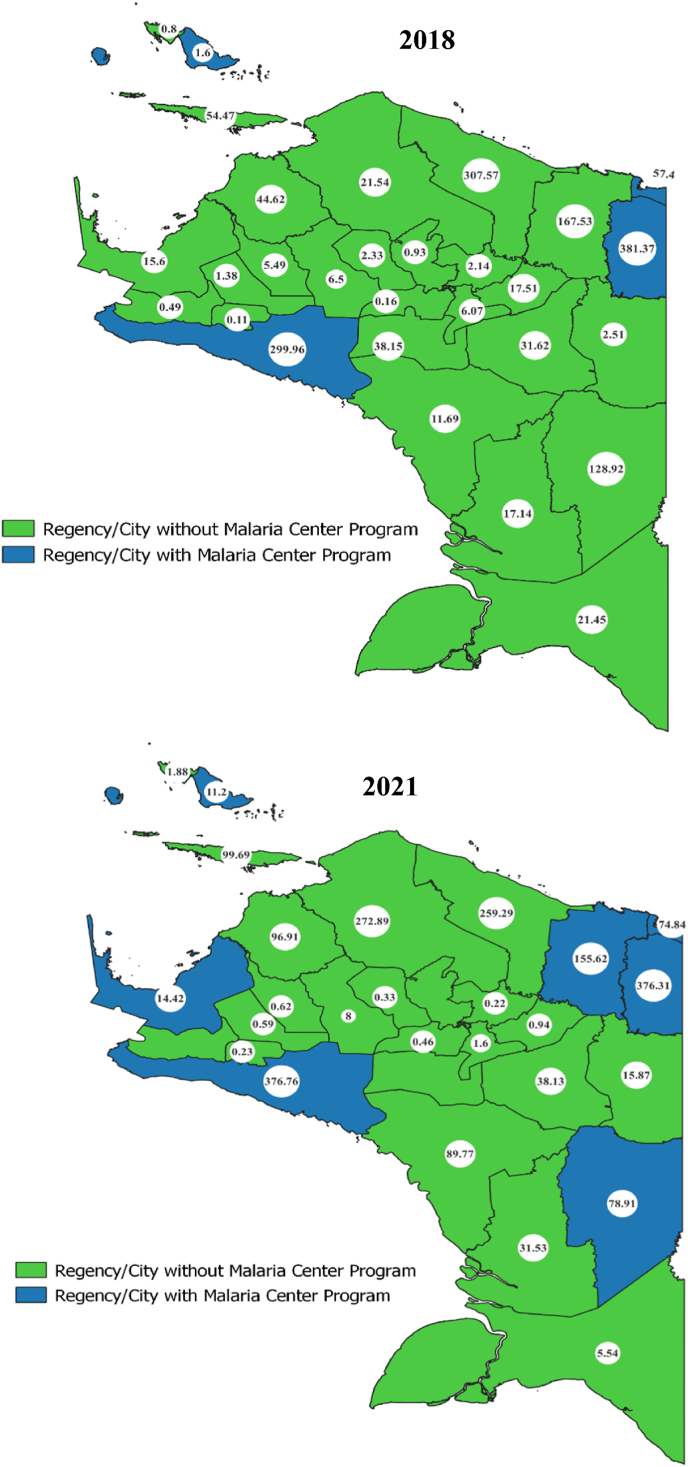
Map of the distribution of malaria centre programs in Papua Province 2018 and 2021 (Directorate General of Disease Prevention and Control and Papua Provincial Health Office).

According to Fig. 3, in 2018, 4 regencies/cities in Papua Province implemented the malaria centre program: Biak Numfor Regency, Mimika Regency, Keerom Regency, and Jayapura City. By 2021, this program expanded to 3 more regencies: Jayapura District, Boven Digoel, and Nabire. This expansion reduced malaria incidence in Jayapura Regency, Keerom Regency, Boven Digoel Regency, and Nabire Regency in 2021. However, some regions, like Mimika Regency, Biak Numfor Regency, and Jayapura City, saw an increase in malaria cases, highlighting the complexity of malaria in these regions. Despite the early adoption of the program in 2013, the rise in cases in 2021 indicates a need to reevaluate its implementation.

### 3.4 Analysis of economic conditions distribution, malaria centre, and annual parasite incidence in Papua Province

**Fig. 4:**
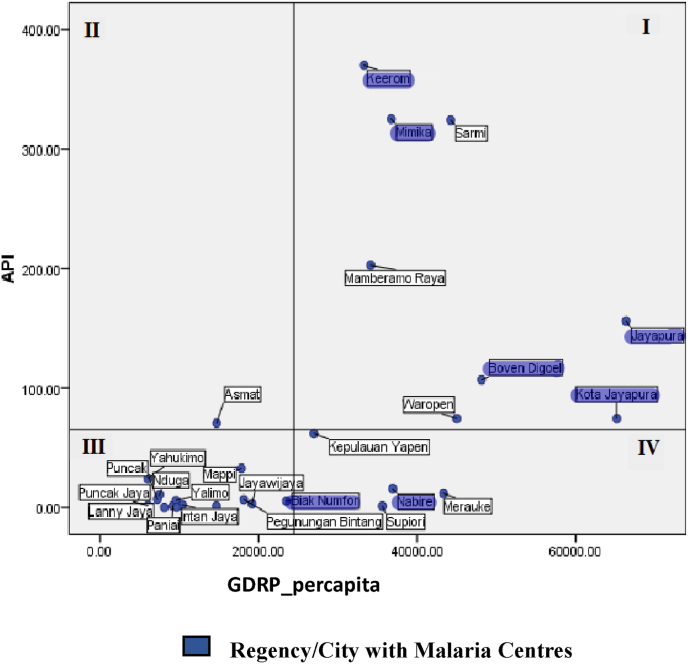
Distribution of regencies/cities by annual parasite incidence, malaria centre, and GRDP in 2018–2021 (Papua provincial health office and Papua central bureau of statistics).

Fig. 4 shows the distribution of regencies/cities in Papua Province based on a quadrant graph comparing the average annual parasite incidence (API) and average GRDP per capita from 2018 to 2021. During this period, the average API was 68.44 cases per 1000 population, and the average GRDP per capita was Rp 24,469,958.

Fig. 4 maps out malaria incidence rates, the presence of malaria centres, and GRDP per capita, leading to the conclusion that regions with high malaria incidence often have higher-than-average GRDP per capita. Notably, 6 regencies/cities with malaria centre programs fall into quadrants I and IV, indicating their GRDP per capita is above the average.

Overall, regions with malaria centres generally have a GRDP per capita above the average and tend to have higher annual parasite incidence. These findings highlight that economic conditions and high annual parasite incidence are key factors in the implementation of malaria centre programs. Regencies with more robust economic activity and better access to healthcare resources are generally more proactive in combating the spread of malaria. The relationship between high malaria incidence rates and regencies with above-average GRDP per capita.

### 3.5 Fixed effects analysis

Fixed effects estimation allows for time-based comparisons and automatically adjusts for characteristics that remain constant during the study [18,22].To enhance analytical efficiency, using fixed effects with cluster-robust standard errors is recommended [16]. Researchers often use "robust" standard errors to ensure accurate statistical inference when some regression assumptions are violated, with the covariance matrix estimators by Huber (1967), Eicker (1967), and White (1980) being widely recognized [23].

The fixed effects estimation results in Table 2 indicate that the malaria centre program has a significant impact on reducing malaria incidence in Papua Province at the 1 % significance level. Specifically, the program reduces malaria incidence by 31.81 cases per 1000 population, which falls within the high endemicity category I [24]. This demonstrates that the malaria centre program has a substantial effect on decreasing malaria cases in Papua Province.

**Table 2:** Fixed effects estimation results.

Variables	(Fixed Effects Cluster Robust)
	Annual Parasite Incidence
Malaria centre	−31.815∗∗∗
	(9.167)
**Health Facilities**	
Hospital ratio	−1155.346
	(1113.133)
Health centre ratio	−430.695∗∗∗
	(128.936)
**Socio-economic Characteristics**	
Primary school enrollment rate	1.012
	(2.084)
Junior high school enrollment rate	−2.451
	(4.198)
Senior high school enrollment rate	−0.388
	(1.411)
Log GRDP per capita	194.101
	(120.104)
Log population size	142.449
	(115.530)
Region	−0.673
	(15.631)
**Geographic Characteristics**	
Log area (km^2^)	4.076
	(4.417)
Log altitude (masl)	15265.145
	(17243.963)
FE.Years	Yes
Constant	−79702.322
	(85302.550)
Observation	111
R-square within	0.188

Cluster robust standard errors in parentheses, ∗*p* < 0.10, ∗∗*p* < 0.05, ∗∗∗*p* < 0.01.

The ratio of community health centres significantly reduces malaria incidence by 430,695 cases, emphasizing their critical role in malaria control. While hospitals don't show a significant direct impact, increasing the hospital ratio by one unit can reduce malaria incidence by 1155 cases per 1000 population. This underscores the importance of healthcare facilities in combating malaria in Papua Province.

Socioeconomic control variables, such as GRDP per capita, school enrollment rates (elementary to high school), population size, and region, do not significantly impact malaria incidence in Papua Province. Similarly, geographical factors like area size and altitude also show no significant effect.

## 4 Discussion

The fixed effects estimation shows that the malaria centre program significantly reduces malaria incidence in Papua Province by 31.81 cases per 1000 population, placing it within the high endemicity category I. The malaria centre program effectively reduced malaria cases in North Maluku [14,12].

In-depth interviews reveal that the malaria centre program in a Papua Province district employs various vector control strategies, including indoor residual spraying (IRS), periodic fogging, mosquito breeding site elimination, insecticide-treated bed net distribution, and mass blood surveys in high-incidence areas. Malaria cases are systematically recorded at community health centres. However, limited coverage hampers public awareness efforts.

Malaria control programs in Africa, like using long-lasting insecticidal nets (LLINs) and indoor residual spraying (IRS), advocate for combination approaches to reduce malaria cases [6], and are believed to have a similar impact in reducing malaria incidence in Papua Province. Malaria control programs using house screening interventions reduced the probability of malaria occurrence in Zambia [6]. Additionally, the PAMAFRO program, which employed a combination of interventions, including LLIN distribution, increased case detection, and malaria case management in Peru, successfully reduced malaria incidence [10].

The malaria program in Papua Province has reduced malaria incidence, but challenges persist, with no regencies achieving elimination. Similarly, Das et al. (2018) found that while reactive case detection (RCD) helps reduce transmission in Zanzibar, additional measures are needed for full elimination [8]. Regions must show no indigenous malaria cases for three consecutive periods to achieve elimination [5].

The epidemiological characteristics of malaria in Papua Province demonstrate significant diversity and uniqueness. This underscores that a universal approach to malaria control and elimination may not be optimally effective, highlighting the need for additional focus on interventions tailored to specific spatial contexts [5]. Despite adopting the malaria centre program since 2013, Mimika Regency struggled to reduce high malaria incidence, reaching 376.76 cases per 1000 in 2021, the highest in Papua Province. In-depth interviews revealed that some interventions were suboptimal.

The distribution of insecticide-treated bed nets by the malaria centre program has been suboptimal, often deemed irrelevant due to local weather conditions, and thus underutilized. Similarly, in Ghana, bed nets did not significantly reduce malaria cases due to the high-risk environment [7]. Monroe et al. (2015) attribute the ineffectiveness of long-lasting insecticidal nets to children's behaviors outside homes during evening hours, night schooling, household chores, and small-scale economic activities [7].

Cross-sector collaboration in malaria control has been ineffective due to limited funding and low engagement from the community and various sectors. Although the program includes private entities like PT. Freeport's Community Health Development (CHD) and non-governmental organizations like the Amungme and Kamoro Community Empowerment Foundation (YPMAK), government involvement is minimal, confined to monitoring, with no active participation from the community in prevention efforts.

Three interviewees indicated that the lack of cross-sectoral and community involvement in malaria eradication programs is due to several factors. These include inadequate advocacy by the malaria centre program implementers with government and community stakeholders, low awareness about engaging in malaria control efforts, and the perception that malaria is solely the responsibility of the health department. Partnerships are crucial factors in driving the success of malaria eradication programs, as these efforts require collaborative efforts across various sectors that should be involved.

The suboptimal performance of the malaria centre program in Papua Province mirrors that in North Maluku Province. Despite successfully reducing malaria incidence, there was a subsequent increase of 15.29 % in the following year. This increase was attributed to the ineffectiveness of mosquito vector control. Furthermore, the performance of the malaria centre is also suboptimal due to inadequate regulatory support, limited stakeholder understanding, incomplete advocacy efforts, and the absence of programs involving relevant sectors [12].

Efforts to combat malaria face major challenges, including limited cross-sectoral engagement due to insufficient advocacy, high treatment costs, and inadequate program funding. Public awareness and understanding of prevention measures are low, contributing to high recurrence rates and ineffective use of bed nets. In Papua, most cases involve *Plasmodium vivax*, which is challenging to control due to its efficient transmission at low parasitemia, low diagnostic sensitivity, and a dormant liver stage that causes relapses and sustained transmission [5].

Interview findings indicate that the malaria centre program in Mimika Regency has not significantly reduced malaria incidence, despite some signs of fewer cases. To enhance effectiveness, public awareness campaigns and prevention banners should be intensified, with better use of funds. Accurate data collection, private sector involvement, and exploring alternatives to bed net distribution are also essential for expanding prevention efforts.

Efforts to prevent and control malaria depend heavily on health facilities, particularly community health centres and hospitals. Based on fixed effects estimation results, the health facility control variables indicate that the ratio of community health centres significantly reduces malaria incidence. Meanwhile, the hospital ratio, although not statistically significant in reducing malaria incidence, shows a potential decrease of 1155.346 cases per 1000 population. These findings underscore the role of health facilities in lowering malaria incidence in Papua.

According to interviews with malaria centre programme implementers and community members, community health centres play a vital role in malaria prevention, diagnosis, and case management efforts. These centres function not only as examination and treatment facilities but also actively engage in community education regarding malaria prevention. In contrast, hospitals have a specialized role in providing medical interventions for malaria patients requiring advanced care. Furthermore, in decisions related to malaria elimination, both health centres and hospitals are involved in managing outbreaks marked by an increased incidence of illness or death in a region [25].

Malaria control programs not only impact the reduction of malaria incidence but also bring about other effects such as decreased mortality rates, increased school participation, improved community economic status, and enhanced workforce productivity [6,11,26].

The Malaria Centre plays a strategic role in malaria control efforts in Papua Province, which remains the highest contributor to malaria cases in Indonesia. The program is implemented through a cross-program and cross-sectoral approach to strengthen collaboration in curbing disease transmission and supporting the achievement of national malaria elimination targets. This approach can also be applied in other highly endemic areas as a strategy to accelerate malaria elimination across Indonesia.

Beyond the role of the Malaria Centre, healthcare facilities such as hospitals and community health centres are essential in sustaining malaria control efforts. Their responsibilities span from health promotion and disease prevention through public education and awareness campaigns to early detection via microscopy and appropriate clinical treatment. The effectiveness of these services largely relies on skilled personnel, particularly laboratory staff like microscopists and cross-checkers who ensure diagnostic accuracy. Strengthening their capacity through regular training and adequate logistical support is therefore crucial to improving malaria surveillance and achieving national elimination targets.

## 5 Conclusion

The Malaria Centre program in Papua Province has played a significant role in reducing malaria incidence. However, its impact remains limited by suboptimal interventions, inadequate cross-sector collaboration, insufficient community involvement, and logistical challenges. Similar control strategies in Africa and Peru demonstrate the effectiveness of integrated approaches, suggesting the need for context-specific solutions in Papua. Strengthening community health centres, enhancing public awareness, and improving intersectoral coordination are critical for sustainable malaria reduction. Addressing these gaps, alongside more effective use of resources and regulatory support, is essential for achieving elimination goals in Papua and other endemic regions of Indonesia.

In short, based on the in-depth analysis and discussions above, the study concludes:

1. The Malaria Centre program has had a significant impact on reducing malaria incidence in Papua Province. Its implementation has successfully decreased malaria cases in several districts, including Keerom, Nabire, Boven Digoel, and Jayapura.
2. In-depth interviews indicate that several interventions by the Malaria Centre have not achieved optimal outcomes. Specifically, the distribution of insecticide-treated nets has not had a significant effect on reducing malaria cases [7]. Moreover, cross-sector collaboration has been ineffective, marked by limited funding and low community engagement, largely due to insufficient advocacy by the Malaria Centre toward both the government and the public.
3. Primary health care facilities, especially community health centres, have played a crucial role in lowering high malaria incidence rates, while hospitals have had a lesser impact in this regard.

## 6 Implications

Based on the findings, the following implications are proposed:

1. The Malaria Centre program has successfully reduced malaria incidence in Papua; however, the target of elimination has not yet been achieved. To accelerate malaria elimination, it is essential to expand the program's coverage to high-endemic areas, accompanied by strengthened surveillance systems and enhanced capacity of health personnel.
2. Strengthening cross-sectoral engagement through regular coordination forums involving key stakeholders, such as health offices, local governments, and civil society organizations, is crucial for developing an integrated malaria control strategy. Advocacy should prioritize enhancing political commitment and securing increased local funding. Furthermore, current interventions, including insecticide-treated nets, should be reassessed, with alternative methods like house screening proven effective in Zambia [6] considered for implementation in high-endemic areas of Papua.
3. Enhancing community health centres through targeted training and equitable access to diagnostics and treatment is vital for malaria control. Simultaneously, hospitals must function as effective referral units by improving infrastructure, workforce capacity, and integrating case management into regional health planning.

## 7 Limitation

The research data, including annual parasite incidence (API), is available only from 2018 to 2021. For a more comprehensive analysis, data from before the malaria centre program's implementation in Papua Province (starting in 2013) should be considered. This study's in-depth interviews were conducted in a single district, limiting the findings to that area's perspective and making them insufficient to represent the program's implementation across the province. Additionally, key malaria-related data, such as climate variables (rainfall, temperature, humidity) and environmental factors (swamps, waterlogging, dense vegetation), are not specifically available at the district/city level in Papua Province.

## Disclosure statement

No potential conflict of interest was reported by the author.

## Appendix

To address potential bias due to missing data in several districts/cities, a robustness check was performed; the results are reported in Supplementary 1.
