# Supplementary material for: The impact of the malaria centre program on malaria incidence in Papua Province
Source: Public Health Pract (Oxf). 2025 May 15;9:100625. doi: 10.1016/j.puhip.2025.100625 (PMC12143789; doi:10.1016/j.puhip.2025.100625)
Supplement: Multimedia component 1 [file mmc1.docx]

## **Robustness Check**

Table 1 Robustness Check Results

|  | **(1)** | **(2)** | **(3)** | **(4)** |
| --- | --- | --- | --- | --- |
| **Variables** | **API** | **API** | **API** | **API** |
| Malaria center | -30.615^**^ | -28.055^**^ | -28.136^***^ | -31.815^***^ |
|  | (12.763) | (10.338) | (9.045) | (9.167) |
| **Health Facilities** |  |  |  |  |
| Hospital ratio |  | 819.398 | -1119.225 | -1155.346 |
|  |  | (1065.762) | (1124.129) | (1113.133) |
| Health center ratio |  | 385.783^***^ | 424.037^***^ | -430.695^***^ |
|  |  | (138.707) | (113.379) | (128.936) |
| **Socio-economic Characteristics** | | |  |  |
| Primary school |  |  | 0.559 | 1.012 |
| enrolment rate |  |  | (2.255) | (2.084) |
| Junior high school |  |  | -3.319 | -2.451 |
| enrolment rate |  |  | (4.019) | (4.198) |
| Senior high school |  |  | -0.408 | -0.388 |
| enrolment rate |  |  | (1.365) | (1.411) |
| Log GRDP per capita |  |  | 196.941 | 194.101 |
|  |  |  | (117.063) | (120.104) |
| Log population size |  |  | 143.128 | 142.449 |
|  |  |  | (113.107) | (115.530) |
| Region |  |  | 0.789 | -0.673 |
|  |  |  | (15.521) | (15.631) |
| **Geographic Characteristics** | | |  |  |
| Log area (km^2^) |  |  |  | 4.076 |
|  |  |  |  | (4.417) |
| Log altitude (masl) |  |  |  | 15265.145 |
|  |  |  |  | (17243.963) |
| FE.Year | Yes | Yes | Yes | Yes |
| Constant | 63.842^***^ | 118.690^***^ | -4551.652 | -79702.322 |
|  | (5.610) | (16.971) | (2983.829) | (85302.550) |
| Observation | 111 | 111 | 111 | 111 |
| R-square within | 0.092 | 0.138 | 0.180 | 0.188 |

Standard errors in parentheses, ^*^ *p* < 0.10, ^**^ *p* < 0.05, ^***^ *p* < 0.01

Robustness checks are essential to address the potential bias that may result from data imbalance (Cirera, et al. 2022). In this study, malaria incidence data for 2021 was missing for several regencies. To mitigate the potential bias caused by this data imbalance, the authors conducted a series of robustness checks.

Table 1 presents 4 research models that analyze the relationship between annual parasite incidence and the malaria center program, including various control variables. The estimation results from all 4 models indicate that the malaria center program consistently and significantly reduces malaria incidence, although there are minor variations in the coefficient values due to the inclusion of different control variables. These findings enhance the reliability of the study by confirming that the impact of the malaria center program on reducing malaria incidence remains robust and is not substantially influenced by the control variables incorporated into the analysis.
